# Risk probability and influencing factors of stroke in followed-up hypertension patients

**DOI:** 10.1186/s12872-022-02780-w

**Published:** 2022-07-24

**Authors:** An-le Li, Ying Ji, Shuai Zhu, Zhi-hao Hu, Xue-jin Xu, Ya-wei Wang, Xin-zhi Jian

**Affiliations:** Jiading District Center for Disease Control and Prevention, Shanghai, China

**Keywords:** Hypertension, Stroke, Risk probability, Factors, Cox regression, Cohort

## Abstract

**Objective:**

To explore the risk probability and main influencing factors of stroke in followed-up hypertension patients through the analysis of long-term followed-up cohort data.

**Methods:**

The method of followed-up observation cohort was used to collect the information of 168,417 followed-up hypertension patients from 2002 to 2020 in Jiading District in Shanghai. Kaplan–Meier method was used to analyze the risk probability of stroke complications in long-term followed-up HTN patients, and the influencing factors were analyzed by Cox proportional risk model.

**Results:**

Among 168,417 followed-up hypertension patients, 11,143 cases had suffered stroke, and the cumulative incidence rate of stroke was 6.62% (male was 6.87%, female was 6.37%). With the extension of the hypertension years, the cumulative risk probability of stroke in HTN patients would continue to increase and the interval was not equidistant. The total cumulative risk probability of stroke in HTN patients was 78.9% (male was 91.0%, female was 70.7%). During the period of hypertension, the risk occurring probability of stroke was not fixed, but fluctuating. There were 4 onset peaks, which were in 8 years (probability was 4.2%), 15 years (probability was 14.0%), 22 years (probability was 6.0%) and 26 years (probability was 13.9%). The highest risk probability of male patients was in 26 years (probability was 23.1%), and the second peak was in 15 years (probability was 15.6%). The highest risk probability of female patients was in 15 years (probability was 12.9%), and the second peak was in 26 years (probability was 8.7%). The risk probability of different gender, BP grade and BMI was different, the male were at higher risk than the female, stage 3 HTN was higher than stage 2 and stage 1 HTN, obese people and underweight people were at higher risk than those who have normal weight. The main factors closely related to the occurrence of stroke complications were age (RR = 2.917, *p* < 0.001), body mass index (RR = 1.654, *p* < 0.001), family history of stroke (RR = 1.386, *p* < 0.001) and blood pressure grade (RR = 1.148, *p* < 0.001).

**Conclusion:**

The risk probability of stroke among hypertension patients was high in followed-up hypertension patients (total 78.9%, male 91.0%, female 70.7%), and would continue to increase disproportionately during period of hypertension (4 different onset peaks). With the persistence of hypertension, the risk probability of stroke would increase continuously. Multivariate Cox regression analysis showed that male patients, patients with HBP, abnormal BMI and positive family history were main factors closely related to the occurrence of stroke complications.

## Introduction

Stroke is an acute cerebrovascular disease caused by many factors, and it is the first cause of death in China and the second largest cause of death in the world [1–3]. Hypertension, diabetes, dyslipidemia, cardiovascular disease and unhealthy lifestyle are all risk factors of stroke, among which hypertension is recognized as the main modifiable risk factor of stroke [4–6]. The guidelines of the American Stroke Association also believe that controlling the risk factors of stroke is an effective prevention and control strategy for stroke [7]. Hypertension patients were prone to having stroke, but not all hypertension patients would have stroke, and previous community population observation results showed that different hypertension patients have different epidemic characteristics and prevalence probability of stroke. In the followed-up population of hypertension patients, ischemic stroke was the most important type, rather than hemorrhagic stroke [8].

The main outcome of hypertension is stroke. Untreated hypertension increases the risk of stroke seven times [9]. Hypertension is present in 70% of patients with intracerebral hemorrhage [9–12]. Different types of hypertension are associated with different types of stroke, an earlier antihypertensive treatment trial with DBP (≥ 90 mmHg) as the inclusion standard showed that every 5 mm Hg reduction in DBP could reduce the risk of stroke and ischemic heart disease by 40% and 14% respectively [9]. The antihypertensive treatment trial of isolated systolic hypertension showed that the reduction of SBP by 10 mmHg and DBP by 4 mmHg could reduce the risk of stroke and ischemic heart disease by 30% and 23% respectively [9, 10]. There are many literature reports on hypertension and stroke at present, but there are few reports from the perspective of risk probability and based on a large sample population in the community.

After understanding the relationship between hypertension and stroke and the association with different types of hypertension and stroke, it is necessary to further explore the risk probability of stroke in hypertension, is it 100% or low probability? This study mainly explored from the method the risk probability of stroke in primary hypertension patients and the specific value of the risk probability and main influencing factors of stroke in followed-up primary hypertension patients. In order to better observe and explore the risk probability and risk factors of stroke in hypertension patients, the data of a long-term follow-up cohort of hypertensive patients in Jiading district in Shanghai was used in this study, and the risk probability and influencing factors of stroke in followed-up hypertension patients was analyzed and explored. We hope that these results can provide some help for the prevention and control of stroke in community in the future.

## Methods

### Data sources

All patients (Note: hypertension patient in this article is primary hypertension) were from the Hypertension Follow-up Management System in Jiading district in Shanghai China. The Follow-up Management System of hypertension have been formally established and implemented since from 2002. The Hypertension Followed-up Management System Database records the medical information of all registered and followed-up hypertension patients. According to the guidelines and policies of hypertension prevention and control, the hypertension patients in communities were registered and followed up, family doctors and public health personnel in the community health service center are responsible for the registration, follow-up and management of hypertension patients in the community, the center for disease control and prevention is responsible for the formulation of technical scheme, the training of personnel and the quality control of follow-up data. All patients had primary hypertension. All medical followed-up information of patients must be truthfully recorded in the system database by followed-up doctors. A total of 168 417 hypertension patients were entered into the database of the observation as the deadline of September 30, 2020. All about stroke occurred in followed-up patients must be recorded in detail. Stroke must be diagnosed and confirmed by a senior hospital. This study was approved by Science and Technology Commission, Health Commission and CDC in Jiading district in Shanghai (No JDCDC-2020-0036). All methods were carried out in accordance with relevant guidelines and regulations.

### Data collection

The registered information and followed-up records of hypertension patients were recorded in the system, mainly including the date of birth, gender, ID number, residential address, occupation, education level, family history, date of establishment, termination date and reasons for termination, blood pressure and blood pressure at each follow-up, history of stroke (type of stroke, diagnostic date and diagnostic hospital), lifestyle (smoking, drinking and physical activity) and so on. Considering the integrity and continuity of the data, some incomplete data and later supplementary variables were not included in this study. The date of occurrence of stroke in hypertension patients must be later than the date of registration. If the date of onset of stroke was earlier than or close to the date of registration, this patient data would not be included.

### Definition and classification

The diagnosis of stroke was based on the clinical diagnostic criteria (corresponding clinical symptoms plus positive results of head CT or MRI) [11–13]. The specific diagnosis was made by the secondary and tertiary hospitals, and then the relevant information was collected by family doctors in the community. Stroke mainly included ischemic stroke, hemorrhagic stroke and unclassified stroke in this study. Ischemic stroke included transient ischemic attack, cerebral infarction. Hemorrhagic stroke included subarachnoid hemorrhage, intracerebral hemorrhage and other non-traumatic intracranial hemorrhage.

The measurement and classification of blood pressure and the diagnosis of hypertension were carried out according to the standard of Chinese guidelines for the prevention and treatment of hypertension [13–15]. BMI was classified according to Asian classification standard [16]: underweight (BMI < 18.5), normal (BMI 18.5–22.9), overweight (BMI 23.0–24.9), obesity I (BMI 25.0–29.9), obesity II (BMI 30.0–39.9), obesity III (BMI ≥ 40.0). The diagnosis of hypertension was implemented by advanced clinical hospitals. The hierarchical management of hypertension patients was divided into high-risk, medium risk and low-risk groups according to the blood pressure value, clinical symptoms, exposure risk factors, clinical complications and target organ damage of patients. The follow-up management requirements of different groups are different.

The queue observed was a dynamic queue, and the time when the observed patients entered and exit the queue was inconsistent. Therefore, the observation starting point defined in this paper refers to the time point when patients were diagnosed with hypertension, diagnostic time. Observation end point refers to the time when hypertension patients have expected outcome events (stroke) or withdraw from the observation queue due to loss of follow-up. Follow up time or observation duration refers to the time difference between the end point and the starting point (observation end point minus observation starting point).

### Statistical analysis

According to the deadline, all the recorded data in the hypertension follow-up management system before the deadline were exported to the Microsoft Excel database, and then the corresponding logic check, data screening and conversion were carried out. Finally, the sorted database was imported into the SPSS statistics software package (IBM SPSS statistics version 21) for statistical analysis. The mean and standard deviation of age, blood pressure and other quantitative data were calculated. The number and frequency (%) were calculated according to gender, number of patients, etc. The chi-square (X^2^) test was used to compare the data between different groups. The risk probability was analyzed by Kaplan–Meier methods, the related influencing factors were analyzed by Cox regression. The end event was whether the patient had stroke (Yes 1/ No 0), the difference between the end-point time of observation minus the time of diagnosis of hypertension was used as the observation time or survival time (unit: year), no stroke occurred or lost follow-up or withdrawal during the observation period were regarded as censoring data. Select the observation time to enter the "Time", stroke event (Yes 1/ No 0) to enter the "Status", categorical variable to enter the "Covariates" and take the minimum value as the reference group for multi categorical variables, “Methods” select Forward LR. Bilateral test, *p* < 0.05 for the difference was statistically significant.

## Results

### Baseline demographic characteristics

In this study, 11,143 cases had developed stroke before the deadline among 168,417 followed-up hypertension patients in Jiading district in Shanghai China. The ratio of stroke among followed-up hypertension patients was 6.62%. The average followed-up time was 5.83 ± 0.01 years, and the percentile of followed time was respectively: 50% 6.0 years, 75% 8.0 years, 95% 14.0 years.

Among selected variables of registration information, nationality groups cannot be grouped because they all are Han. In the early occupation registration, because the registration was not standardized, it was difficult to classify and did not enter the analysis. Considering the elderly patients basically in low education level group, the educational level was not included in the analysis. The results showed that the difference of stroke in these variable groups was still obvious (*p* < 0.05), through the grouping data comparison of age, BMI, blood pressure, smoking habit and other variables. See Table1.

**Table 1:** The occurrence of stroke in followed-up hypertension patients among variable groups

Variables	Category	Stroke occurred				X^2^	*p*
		Yes		No			
		n	%	n	%		
Sex	male	5636	6.87	76,363	93.13	17.091	< 0.001
	female	5507	6.37	80,911	93.63		
Age	< 30	0	0	285	100	424.276	< 0.001
	30–39	21	0.68	3071	99.32		
	40–49	250	1.70	14,472	98.30		
	50–59	1417	3.29	41,591	96.71		
	60–69	3629	5.93	57,589	94.07		
	70–79	3800	11.79	28,441	88.21		
	≥ 80	2026	14.63	11,826	85.37		
Body mass index	Normal	2860	6.05	44,413	93.95	111.641	< 0.001
	underweight	318	10.0	2895	90.00		
	Overweight	3299	6.82	45,580	93.18		
	obesity I	4028	6.70	56,721	93.30		
	obesity II	602	8.11	6899	91.89		
	obesity III	36	11.84	271	88.16		
Blood pressure	Stage I	6074	6.27	90,732	93.73	126.722	< 0.001
	Stage II	3522	6.62	49,687	93.38		
	Stage III	1547	8.41	16,855	91.59		
Smoking habit	Every day	2118	5.97	33,330	94.03	213.188	< 0.001
	Occasionally	255	5.21	4638	94.79		
	Have quit	976	9.92	8864	90.08		
	Never	7794	6.59	110,442	93.41		
Drinking habit	Never	8966	7.00	119,104	93.00	149.764	< 0.001
	Occasionally	1353	5.40	23,695	94.60		
	Frequently	622	6.04	9680	93.96		
	Every day	202	4.04	4795	95.96		
Physical activity	Rarely	1150	4.14	26,614	95.86	451.411	< 0.001
	Occasionally	2271	6.66	31,840	93.34		
	Frequently	2892	6.34	42,742	93.66		
	Every day	4830	7.93	56,078	92.07		
Family history of HTN	No	6573	7.60	79,961	92.40	276.587	< 0.001
	Yes	4570	5.58	77,313	94.42		
Family history of stroke	No	10,676	6.63	150,250	93.37	11.854	0.001
	YES	467	6.23	7024	93.77		
BP control level	Average	10,071	6.72	139,873	93.28	14.257	0.001
	Good	435	5.68	7226	94.32		
	Excellent	637	7.01	8446	92.99		

### Risk probability of stroke in HTN patients

**Fig. 1:**
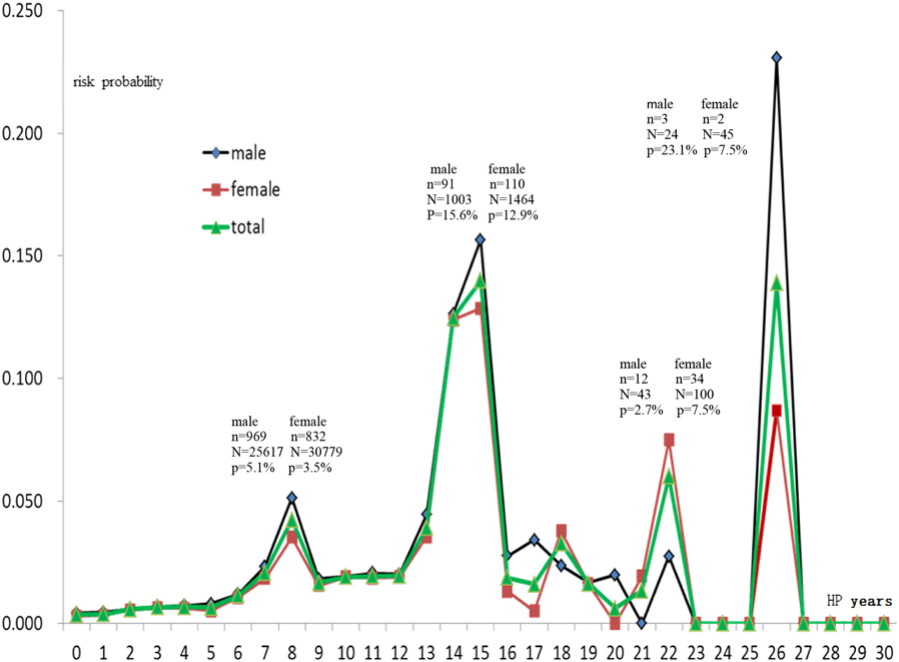
Risk occurred probability of stroke in hypertension patients at different time

To explore the change of risk probability of stroke in hypertension patients during period of hypertension, the hypertension time (unit: year) was selected as the horizontal axis of time, and the occurrence of stroke (including ischemic stroke, hemorrhagic stroke and unclassified stroke) was defined as the event state, and Kaplan–Meier method was used to analyze the risk occurred probability of stroke. The calculated risk probability of each time point was made as a longitudinal axis and a dynamic curve was drawn. See Fig. 1. The result of Fig. 1 showed that the risk probability of stroke in HTN patients was not fixed during the whole hypertension period, but fluctuating. There were 4 peak onset periods, which were in 8 years (probability 0.042, 4.2%), 15 years (probability 0.140, 14.0%), 22 years (probability 0.060, 6.0%) and 26 years (probability 0.139, 13.9%). The highest risk of male patients was in 26 years (probability 0.231, 23.1%), and the second peak was in 15 years (probability 0.156, 15.6%). The highest risk of female patients was in 15 years (probability 0.129, 12.9%), and the second peak was in 26 years (probability 0.087, 8.7%). The risk probability of stroke was different between male and female patients, and male were higher than female.

**Fig. 2:**
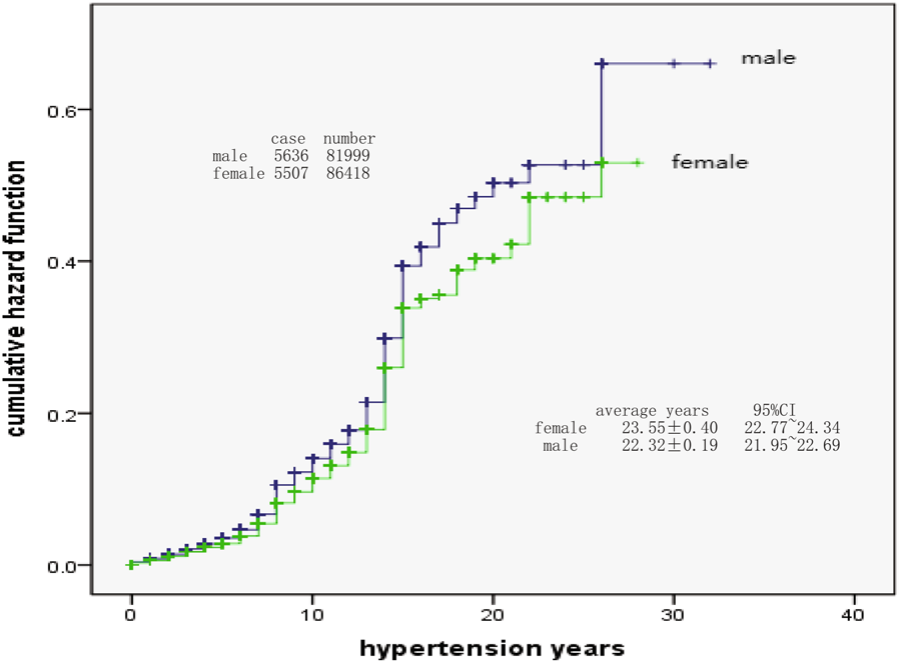
Sex and hazard of stroke

The result of Fig. 2 showed that cumulative risk probability of stroke in male and female patients would continue to increase with the time extension of hypertension years, and the proportion of increase was not equidistant. The cumulative risk probability was higher in male than that in female (x^2^ = 113.570, *p* < 0.001). By calculating the cumulative risk probability of stroke in hypertensive patients of different genders, the results showed that the total cumulative risk probability of stroke in hypertension patients was 0.789 (78.9%), and male was 0.910 (91.0%), female was 0.707 (70.7%).

**Fig. 3:**
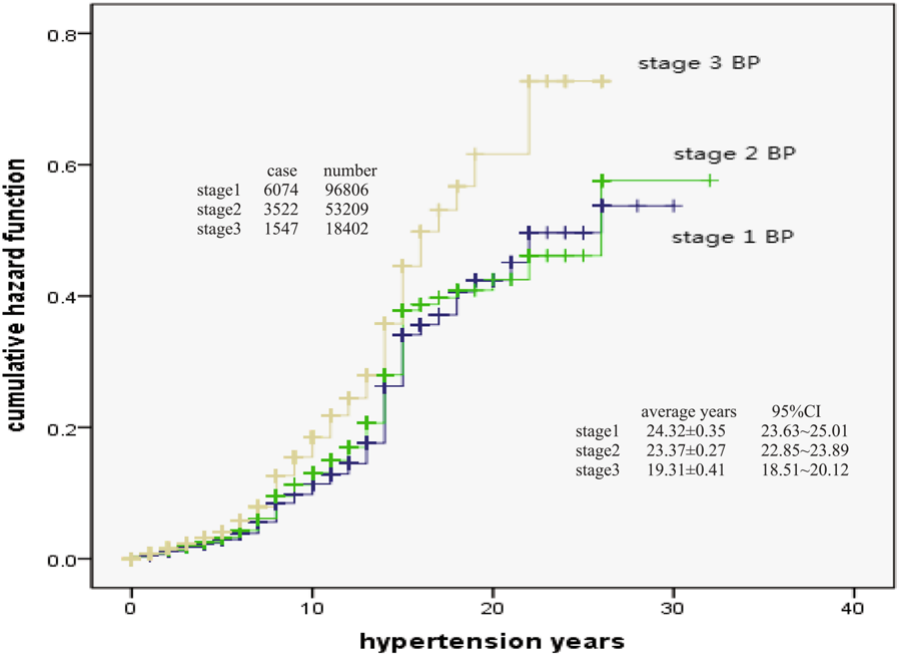
Blood pressure and hazard of stroke

The result of Fig. 3 showed that the cumulative risk probability of stroke in different blood pressure (BP) patients would continue to increase with HTN time. The cumulative risk probability was obviously higher in stage 3 (SBP ≥ 180 or/and DBP ≥ 110 mmHg) patients than that in stage 2 BP (SBP: 160–179 or/and DBP: 100–109 mmHg) and stage 1 BP (SBP: 140–159 or/and DBP: 90–99 mmHg) patients (x^2^ = 189.139, *p* < 0.001). The risk probability of stage 2 BP patients was basically similar to that of stage 1 BP patients at the beginning, but the risk of stroke will suddenly increase after 22 years. It is unclear why. In patients, it may be superimposed or caused by some other factors, which needs further observation and research.

**Fig. 4:**
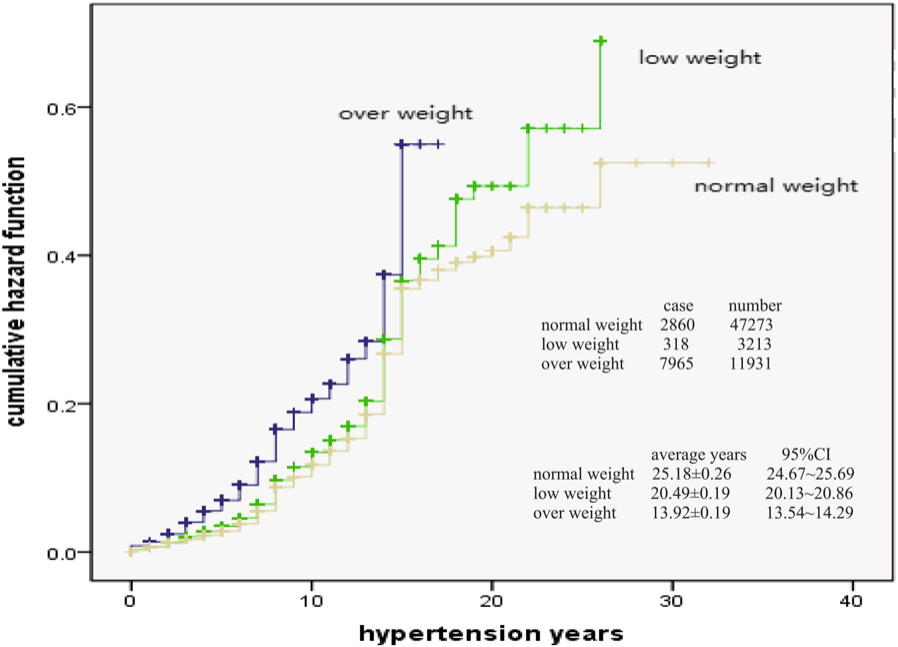
Body mass index and hazard of stroke

Figure 4 showed that the cumulative risk probability of stroke in different body mass index (BMI) patients would continue to increase with hypertension time. The cumulative risk probability was higher in underweight and overweight patients than that in normal weight patients (x^2^ = 86.431, *p* < 0.001).

**Fig. 5:**
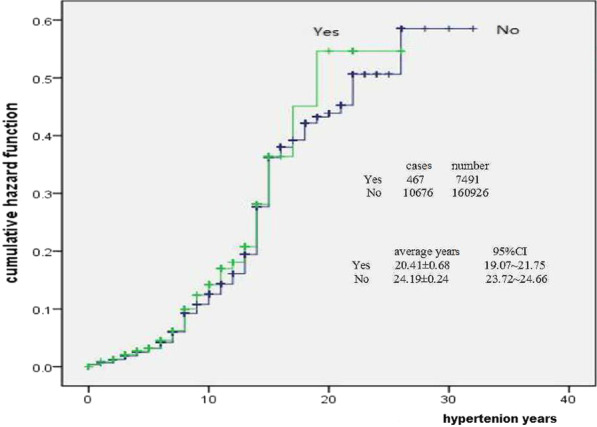
Family history of stroke and hazard of stroke

The result of Fig. 5 showed that the cumulative risk probability of stroke in different family history of stroke patients would continue to increase with hypertension time. But the cumulative risk probability was no difference between hypertension patients with positive and negative family history of stroke (x^2^ = 2.432, *p* = 0.119).

**Fig. 6:**
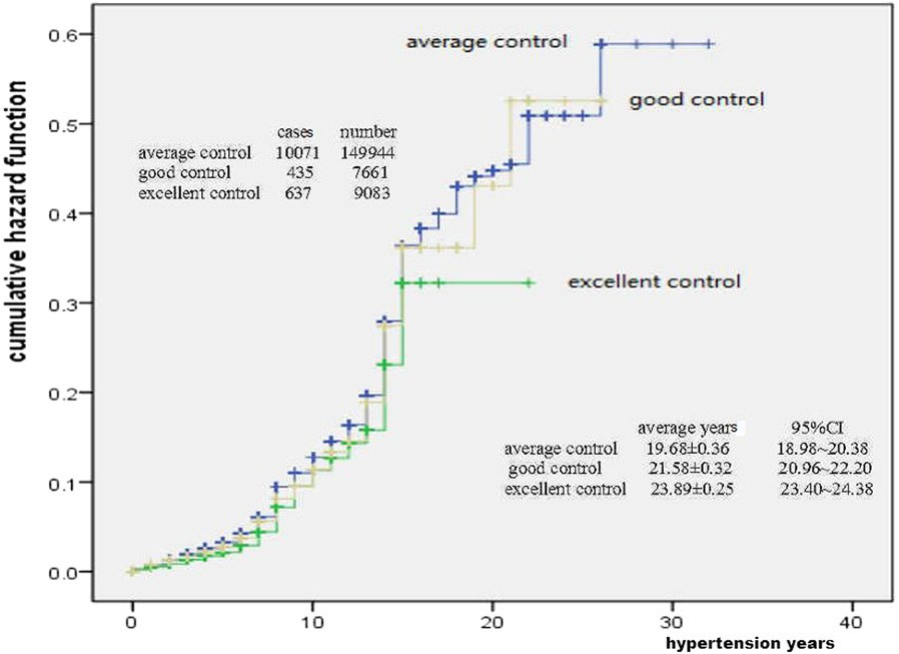
Controlled lever of blood pressure and hazard of stroke

The result of Fig. 6 showed that the cumulative risk probability of stroke in different controlled level of blood pressure would continue to increase with hypertension time. The cumulative risk probability was obviously higher in average (or poor) controlled level ( below 50% of blood pressure records ≤ 140/90 mmHg) and good controlled level (50–75% of blood pressure records ≤ 140/90 mmHg) patients than that in excellent controlled level (75% or more of blood pressure records ≤ 140/90 mmHg) patients (x^2^ = 14.257, *p* < 0.001).

### Influencing factors of stroke

In this study, some long term followed factors including sex, age, smoking habit, drinking habit, physical activity, body mass index, blood pressure grad and family history (including family history of hypertension and stroke) were selected. Cox regression analysis was used to explore the influence of these factors on the occurrence of stroke complications. From the results of Cox regression analysis, these factors have different degrees of influence. Sex, age, smoking habit, drinking habit, physical activity, body mass index, blood pressure grad, family history of stroke, family history of hypertension and BP control level were the influencing factors of stroke in hypertension patients (*p* < 0.05), but the direction and degree of these factors were different. At the same time, considering that age, body mass index and blood pressure have a great impact on the occurrence of stroke, in order to better show the impact of different levels of age, body mass index, blood pressure and HP control level, we further made a more detailed analysis of the impact of different levels of them. See Table 2.

**Table 2:** The Cox regression analysis results of influencing factors of stroke on hypertension

	B	SE	Wald	*df*	Sig	Exp (B)	95% CI RR	
							Lower	Upper
Sex	− 0.357	0.024	226.578	1	0.000	0.700	0.668	0.733
Smoking habit	− 0.030	0.010	8.439	1	0.004	0.970	0.951	0.990
Drinking habit	− 0.187	0.018	104.865	1	0.000	0.830	0.801	0.860
Physical activity	− 0.040	0.010	16.278	1	0.000	0.961	0.942	0.980
Family history of stroke	0.305	0.048	39.741	1	0.000	1.357	1.234	1.491
Family history of HTN	− 0.053	0.021	6.555	1	0.010	0.949	0.911	0.988
Blood pressure								
Stage I			183.721	2	0.000			
Stage II	0.385	0.029	181.140	1	0.000	1.168	1.044	1.720
Stage III	0.273	0.031	79.998	1	0.000	2.276	1.717	2.808
Age < 30			1856.195	6	0.000			
30–39	− 5.815	13.532	0.185	1	0.067	0.003	0.000	0.098
40–49	− 3.396	0.707	23.053	1	0.000	0.034	0.008	0.134
50–59	1.914	0.137	193.877	1	0.000	1.148	1.113	1.193
60–69	1.443	0.050	833.269	1	0.000	1.381	1.262	2.261
70–79	0.964	0.027	1247.999	1	0.000	2.588	2.262	3.402
≥ 80	0.531	0.023	537.079	1	0.000	3.858	3.326	4.615
Body mass indexnormal			49.833	5	0.000			
overweight	− 0.300	0.059	26.152	1	0.004	0.741	0.660	0.831
obesity I	0.366	0.059	38.107	1	0.000	1.693	1.067	1.779
obesity II	0.313	0.059	28.218	1	0.000	2.137	1.652	2.821
obesity III	0.194	0.073	7.048	1	0.008	2.824	2.174	3.950
underweight	0.166	0.256	0.417	1	0.018	1.180	1.014	1.950
BP control level								
average control			189.376	2	0.000			
good control	− 0.429	0.036	139.246	1	0.000	0.669	0.621	0.720
excellent control	− 0.282	0.034	66.759	1	0.000	0.651	0.606	0.699

Different blood pressure grades, age groups and body mass index had different effects on the occurrence of stroke. The results of Table 2 showed that different classified RR of them, the higher blood pressure, the older age and the higher body mass index, the higher risk of stroke in hypertension patients. Generally speaking, among these influencing factors, the more obvious factor was major influencing factors of stroke complications were age (merged RR = 2.917), body mass index (merged RR = 1.654), family history of stroke (merged RR = 1.386), blood pressure grad (merged RR = 1.148). BP (blood pressure) control level is a protective factor. The higher the level of blood pressure control (or the better the blood pressure control), the lower the risk of stroke.

## Discussion

The main harm of hypertension lied in its complications. In the incidence spectrum of complications in hypertension patients, the constituent ratio from high to low was cerebrovascular diseases (49.61%), diabetes mellitus (34.94%), heart damage (13.93%), kidney diseases (1.04%), fundus damage (0.30%) and peripheral vascular diseases (0.04%). Cardiovascular and cerebrovascular diseases were the main complications in patients with hypertension in community [17]. In this study, the cumulative incidence rate of stroke in hypertensive patients was 6.62%, the male was 6.87% and the female was 6.37%. The incidence rate of stroke in hypertensive patients was much higher than that incidence rate in general population [1, 3, 18]. Therefore, it was an important link in community stroke prevention and control that strengthen the blood pressure control of patients with hypertension and prevent the occurrence of stroke in patients with hypertension. Jiading District government in Shanghai China began to implement the free medication policy for hypertension patients with rural medical insurance as early as around 2000. Later, due to the cancellation of rural household registration and the implementation of urban population management policy in Shanghai, the free medication policy gradually faded out, but it was still open to poor households. What need to be explained here again was that vast majority of patients of this followed-up were taking antihypertensive drugs, only a few patients were not willing to accept drug treatment. The management of hypertension refers to the hierarchical management of patients according to the blood pressure value and exposed risk factors according to the national guidelines for the prevention and treatment of hypertension in Shanghai China. Medical followed-up information of hypertension patients (including blood pressure value, antihypertensive drug used, height and weight, lifestyle, concurrent diseases, etc.) is recorded in the database of the hypertension followed-up management system. At the end of each year, all patients are evaluated for blood pressure control and re stratified. Every year, the quality control personnel in CDC regularly carry out the quality control of the followed-up authenticity and information compliance rate, and evaluate the standardized management rate, blood pressure control rate and other indicators, and the government will incorporate the quality control index results into the performance appraisal. Doctors are required to strictly require the guidelines to regulate the management of patients, because quality control indicators are directly linked to doctors' income. The proportion of standardized oral antihypertensive drugs in followed-up hypertensive patients was more than 95%. Assuming these patients did not receive antihypertensive treatment, the incidence of stroke might be higher. Based on medical ethics, it was difficult to observe the dynamic changes of patients in the natural original state. In addition, considering the continuity and consistency of data, some important risk factors and variables collected in the later stage were not analyzed in this study. These were the limitations and deficiencies of this study.

At present, there were many reports on the risk probability analysis or prediction of stroke with hypertension, and the methods used include Logistic Regression analysis, Framinghan evaluation method and ESRs score method [19–22]. The results of different methods are different. Kaplan–Meier method was used in this study, which is also called product limit method. It was not only suitable for small sample data, but also suitable for large sample data. In addition, this method could take into account the objects exiting the observation queue or incomplete censored data, so it was more applicable. Through the analysis of long-term observation data, it was found that with the extension of hypertension years, the cumulative risk probability of stroke in hypertension patients would continue to increase, and the increasing distance was not equidistant. The total cumulative occurred probability of stroke in hypertension patients was 0.789 (78.9%), and male was 0.910 (91.0%), female was 0.707 (70.7%). Male was higher than female. The observation results remind us that hypertension patients must control their blood pressure as soon as possible. Although not all patients with hypertension will have stroke complications, the probability of occurrence is relatively high, and with the extension of the course of disease, the risk of stroke will increase, and finally reach a very high risk probability, especially male patients. Male patients are more dangerous than female. From the perspective of risk probability, the cumulative risk probability was obviously higher in stage 3 BP patients than that in stage 2 BP and stage 1 BP patients, higher in underweight (low weight) and overweight patients than that in normal weight patients, and also higher in average (or poor) controlled level and good controlled level patients than that in excellent controlled patients.

The main type of stroke was cerebral infarction in hypertension in Jiading Shanghai, the proportion of ischemic cerebrovascular, hemorrhagic cerebrovascular and unclassified stroke was respectively 71.18%, 5.95% and 22.87% in hypertensive stroke [8]. Based on the small proportion of hemorrhagic stroke, the subtypes of stroke were not calculated separately in the data analysis, which is the lack of rigorous place in this study. The development of hypertension to stroke needs a process, and the length of the process depends on the patient's own body condition and so on. The risk occurred probability of stroke in hypertension patients was not fixed during the whole hypertension years, but fluctuates. There were 4 onset peaks, which were in 8 years (peak value 0.042, 4.2%), 15 years (peak value 0.140, 14.0%), 22 years (peak value 0.060, 6.0%) and 26 years (peak value 0.139, 13.9%). Why did the risk probability of stroke in hypertension patients show four different peaks instead of one-way change, this might be that the blood pressure was not effectively improved, and other risk factors accumulate, resulting in vascular damage to a certain extent, leading to the outbreak of stroke. Other factors might be superimposed. The highest risk probability of male patients was in 26 years (peak value 0.231, 23.1%), and the second peak was in 15 years (peak value 0.156, 15.6%). The highest risk of female patients was in 15 years (peak value 0.129, 12.9%), and the second peak was in 26 years (peak value 0.087, 8.7%). Although the time point of outbreak was the same for male and female, the occurrence probability of both was significantly higher in male than in female. This might be a more risk factor for male exposure than female exposure, there might also be other unknown factors related to gender. In addition, if hypertensive patients did not receive antihypertensive treatment, the risk probability of stroke might be higher, and the peak time point might be more advanced.

Stroke is a group of cerebrovascular circulation disorders caused by various reasons, manifested as focal neurological deficit, and even accompanied by disturbance of consciousness. Because of its sudden onset, it is also called cerebrovascular accident. There were many risk factors for stroke, such as age, heredity, hypertension, heart disease, arrhythmia, diabetes, hyperlipidemia, smoking, drinking, obesity, high salt, high animal oil diet, excessive physical activity, etc. [9, 23–30]. The risk of stroke was higher in the hypertension and diabetes population. From the results of Cox regression analysis, these factors have different degrees of influence (*p* < 0.05). According to the results of the long-term observation of registered hypertension patient, the major influencing factors of stroke complications were age (RR = 2.917), body mass index (RR = 1.654), family history of stroke (RR = 1.386), blood pressure grad (RR = 1.148,), hypertension duration (RR = 1.071) and family history of hypertension (RR = 1.051). By further classifying levels of age, blood pressure and body mass index, the results showed that different blood pressure grades, age groups and body mass index had different effects on the occurrence of stroke. The higher blood pressure, the older age and the higher body mass index was, the higher risk of stroke was in hypertension patients. These results were consistent with the above risk occurred probability of stroke in hypertension patients and literature reports. The influence of sex, smoking habit, drinking habit and physical activity on stroke complications was statistically significant, the lower limit value of RR of were less than 1. This also means that the direction of action of these factors was protective factors or opposite effect. The reason might be affected by the assignment of data classification, for example, male was assigned as 1 and female was assigned as 2 in sex classification, but the risk of male was actually high than that of female. It might also be caused by other factors. This needs further study, but it is undeniable that sex, smoking habit, drinking habit and physical activity are the influencing factors of stroke, which must be paid attention to in community prevention and control.

Atrial fibrillation (AF) is the most common arrhythmia and has significant morbidity. Morphological voltage P wave duration (MVP) ECG score is of great significance in predicting ischemic stroke hospitalization and long-term atrial fibrillation [31]. A score composed of easily measured electrocardiographic variables to identify patients at risk of AF would be of great value in order to stratify patients for increased monitoring and surveillance. It has been reported that abnormal P-wave index is related to the occurrence of atrial fibrillation and ischemic stroke. It has also been reported that atrial fibrillation (AF) can increase the risk of ischemic stroke by about 5 times. We pay attention to this information, but it is a great pity that China's primary medical institutions did not have the ability to carry out these testing services and these data could not be collected in the past hypertension followed-up. The calculation of these indexes requires special analysis software and digital ECG, these analysis results can't be presented in this study.

In short, hypertension patients were prone to stroke, and the total cumulative occurred probability of stroke in followed-up HTN patients was 0.789 (78.9%), and male was 0.910 (91.0%), female was 0.707 (70.7%). The risk probability of stroke among hypertension patients was high and would continue to increasing disproportionately during period of hypertension, outcome of stroke in HTN patients would have four different onset peaks. Male, blood pressure level (stage 3 BP), abnormal weight (underweight and overweight) and blood pressure control level could increase the risk probability of stroke. The results of multivariate Cox regression analysis showed that male patients, patients with high blood pressure, abnormal body mass index and positive family history were high-risk objects of stroke. Paying attention to blood pressure, weight and male are important and effective measure to prevent stroke in community.

## Data Availability

The data that support the findings of this study are available from the Hypertension Follow-up Management System database in Jiading district in Shanghai, but restrictions apply regarding the availability of these data, which were used under license for the current study and thus are not publicly available. The data are, however, available from the authors upon reasonable request and with permission of the Jiading district health committee in Shanghai.

## Abbreviations

HTN: Hypertension
BP: Blood pressure
SBP: Systolic blood pressure
DBP: Diastolic blood pressure
BMI: Body mass index
RR: Relative risk
CI: Confidence interval
